# A worldwide research overview of Artificial Proprioception in prosthetics

**DOI:** 10.1371/journal.pdig.0000809

**Published:** 2025-04-22

**Authors:** Octavio Diaz-Hernandez

**Affiliations:** Escuela Nacional de Estudios Superiores Unidad Juriquilla, Universidad Nacional Autónoma de México, Mexico City, México; University of Pittsburgh School of Medicine, UNITED STATES OF AMERICA

## Abstract

Proprioception is the body’s ability to sense its position and movement, which is essential for motor control. Its loss after amputation poses significant challenges for prosthesis users. Artificial Proprioception enhances sensory feedback and motor control in prosthetic devices. This review provides a global overview of current research and technology in the field, emphasizing feedback mechanisms, neural interfaces, and biomechatronic integration. This work examines innovations in sensory feedback for amputees, including electrotactile and vibrotactile stimulation, artificial intelligence, and neural interfaces to enhance prosthetic control. The methodology involved reviewing studies from Scopus, Web of Science, and PubMed on prosthetic proprioceptive feedback from 2004 to 2024, evaluating sensory feedback research by author, country, and affiliation with a synthesis provided. Countries like the United States and Italy are collaborating to advance global research. The paper concludes with potential developments, such as advanced, user-centered prosthetics that meet amputees’ sensory needs and significantly enhance their quality of life.

## Introduction

Humans rely on sensory systems (touch, proprioception, and vision) to open doors, navigate spaces, and drive. These systems are integral to the closed-loop motor control process. Understanding how tactile, proprioceptive, and visual systems work provides insight into movement control and its limitations in various skill-based activities. Proprioception, a key but often-overlooked sense, informs the brain about the body’s position, movement, and muscle activity, allowing for real-time adjustments during motion, which is crucial for motor control theories [1].

Proprioception relies on specialized receptors, including proprioceptors in muscles, tendons, and joints, essential for this “sixth sense.” Key components are Golgi-tendon organs (monitoring tension at muscle-tendon junctions), muscle spindles (detecting changes in length and tension), and joint receptors (providing information on joint movement). These receptors send signals to the brain via sensory neurons, where the data is processed to understand body movement and position. In motor skills, two types of feedback occur: task-intrinsic feedback from performing a task and enhanced feedback, also known as task-extrinsic or external feedback, which offers additional performance-related insights [2].

Limb amputation significantly impacts an individual’s health and quality of life [3]. Amputees face challenges in movement and tasks due to the loss of sensory feedback, especially proprioception, which is vital for these activities. The critical role of sensory feedback in upper limb prosthetics has been acknowledged since 1999, highlighting amputation’s profound impact on individuals’ lives [4] This led to the proposal of Artificial Proprioception (AP) as a significant advancement in amputee rehabilitation [5]. AP can restore somatosensory feedback for task performance through electronic systems that allow the brain to perceive critical information. AP is the technological replication of the body’s natural ability to sense position, movement, and force. It enhances prosthetic limbs for individuals with limb loss [5]. AP offers real-time sensory feedback through neural interfaces, electrotactile or vibrotactile stimulation, and advanced systems, allowing users to better control and interact with their prostheses [5].

This review aims to find relevant work on AP around the world, focusing on who, where and when the research about this. Also is considered and discussed the technology used, noted advantages, and actions taken to improve the quality of life for prosthesis users.

## Methodology

### Eligibility criteria

Studies will focus on sensory feedback from external devices globally. Keywords for searches are listed below. The target population is amputees with upper and lower limb prostheses.

### Search strategy

Data sources such as Scopus, Web of Science, and PubMed will be used for searches. The search terms are: (a) Proprioception, (b) Sensory feedback, and (c) Prosthesis, with which the basic searches are done with the different search engines. We will set up a filter to retrieve documents from 2004 to 2024.

### Study selection

The selection process will include reviews of titles, abstracts, methodology, findings, conclusions, and future work. Mendeley will serve as the reference manager.

### Data extraction

The variables extracted from the included studies will be the general information of the paper, e.g., authors, country, and affiliation.

## Results

**Fig 1:**
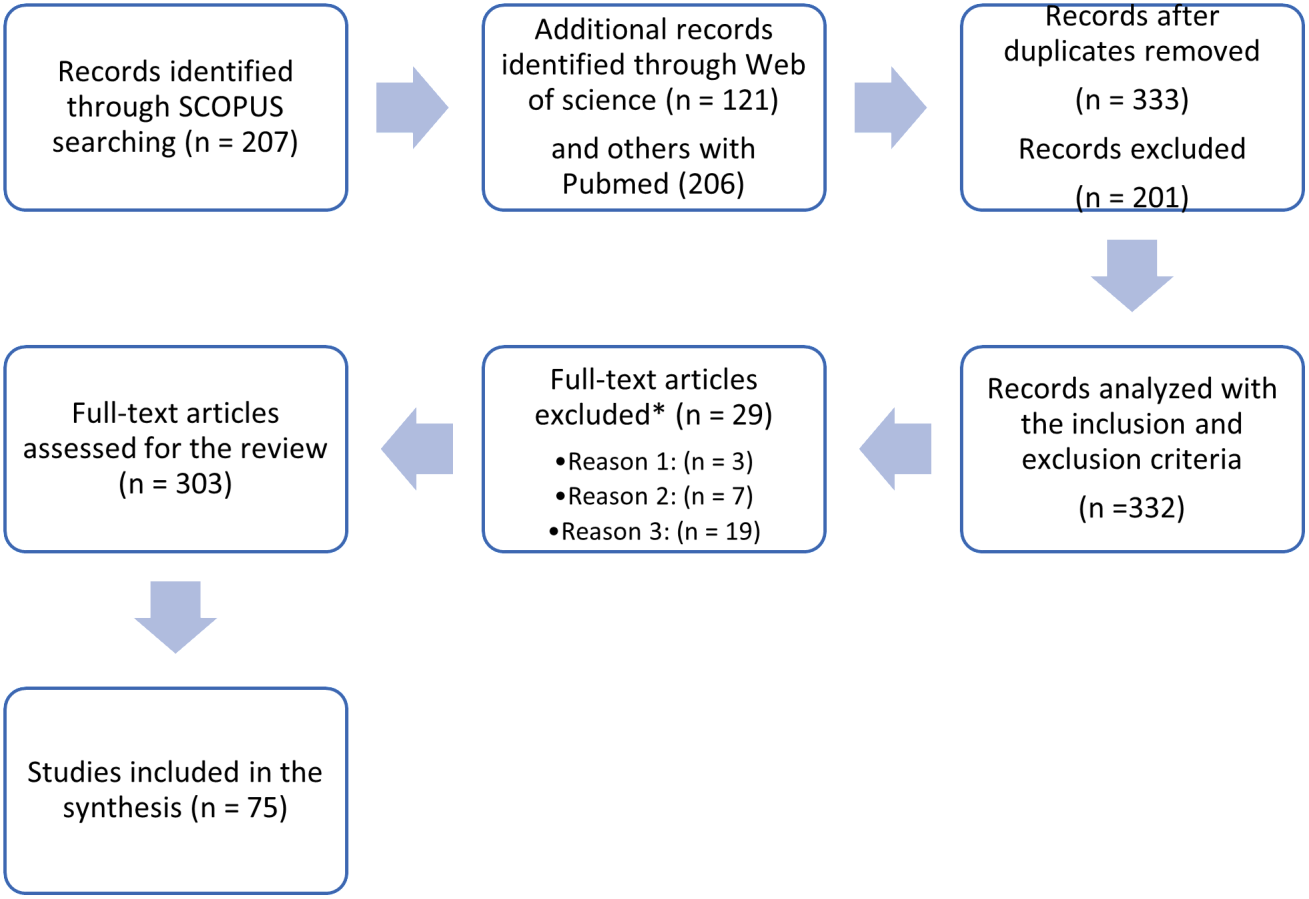
Steps for the study selection. Reasons for exclusion* 1: animal-related studies; 2: other types of prostheses; 3: different areas of study.

The analysis period was 2004-2024. Studies in Scopus, PubMed, and Web of Science were searched; other databases like Nature and SpringerLink yielded repetitive results. Keywords included proprioception, feedback, and prosthesis. Fig 1 shows the study flowchart paper selection.

A total of 534 documents were registered, but 202 duplicates were removed, leaving 332 works in the Mendeley database. The author analyzed the abstracts and titles before reviewing and removing unrelated research.

Twenty-nine documents were removed because they were not related to the topic, e.g., animal studies, ear prostheses, dental prostheses, basic neuroscience studies, arthroplasty, intracortical brain-machine interfaces, biomaterials, song production in birds, and the role of vestibular cues in postural sway. In the end, the review focused on and used 75 documents related to the subject.

### Analysis by the general information

#### By author.

This provides an overview of publications from top authors in sensory feedback research. Table 1 outlines significant biomedical engineering and robotics authors and their contributions, affiliations, and countries. Dustin J. Tyler leads in the United States (U.S.) with 12 publications. Christian Cipriani and Stanisa Raspopovic have 10 publications. Strahinja Dosen in Denmark and Giacomo Valle in Switzerland have nine documents. The U.S. also has Allison M. Okamura and Silvestro Micera from Italy, who have eight publications. Katherine J. Kuchenbecker from the University of Pennsylvania has five papers.

**Table 1:** List of authors with the most significant number of publications.

Authors	Number of documents	Affiliation	Country	Cites
Dustin J. Tyler	12	Department of Biomedical Engineering, Case Western Reserve University, Cleveland, Ohio.	United States of America	[4–15]
Christian Cipriani	10	The BioRobotics Institute, Scuola Superiore Sant’Anna, Pontedera	Italy	[16–25]
Stanisa Raspopovic	10	Neuroengineering Laboratory, Department of Health Sciences and Technology, Institute for Robotics and Intelligent Systems, ETH Zürich, Zürich	Switzerland	[22,26–34]
Giacomo Valle	9	Neuroengineering Laboratory, Department of Health Sciences and Technology, Institute for Robotics and Intelligent Systems, ETH Zürich, Zürich	Switzerland	[21,22,27,28,30–33,35]
Strahinja Dosen	9	Department of Health Science and Technology, Aalborg University, Aalborg	Denmark	[19,20,36–42]
Silvestro Micera	8	The BioRobotics Institute, Scuola Superiore Sant’Anna, Pontedera	Italy	[7,21,22,24,28,43–45]
Allison M. Okamura	8	Department of Mechanical Engineering, Stanford University, Stanford, California	United States of America	[46–53]
Katherine J. Kuchenbecker	5	Department of Mechanical Engineering and Applied Mechanics, University of Pennsylvania, Philadelphia, Pennsylvania	United States of America	[46–50]

Here, we offer a brief overview of the research conducted by Dustin J. Tyler and his team, who have substantially contributed to neural interfaces for prosthetic limbs. Their efforts specifically aim to restore sensory feedback, significantly improving the user experience.

Tyler and colleagues [4] explored neural interfaces for prosthetic feedback, restoring tactile sensations and reducing phantom limb pain for amputees. This nervous system connection enables users to “feel” through prosthetics, enhancing functionality and psychological well-being. In qualitative studies, Tyler and Graczyk (2019) highlighted how restored sensation profoundly enhances users’ emotional experience, improving their quality of life. Building on this, Tyler with Segil in 2020 [7] demonstrated how combining multiple artificial sensory percepts enables users to identify prosthetic hand postures with higher accuracy when sensory feedback mirrors natural anatomical mappings. Page and colleagues (2021) [8] expanded on these findings, showing how electrical stimulation via intraneural interfaces allows users to discriminate between various hand percepts, improving control over bionic arms. Together, these studies reflect the evolving potential of neural interfaces in restoring sensory and motor function in prosthetic users. Bensmaia and colleagues (2023) [7] further reviewed the possibility of non-invasive and invasive technologies to transmit sensory feedback, emphasizing their long-term prospects for restoring independence in amputees.

We present a synthesis from Cristhian Ciprani and collaborators, the second-largest contributor in this review.

A study by Cipriani and Markovic [20] developed a system integrating stereovision to select grasp type and size, while augmented reality provides artificial proprioceptive feedback. The system was tested on healthy subjects, successfully implementing a low-effort, high-level control interface adaptable for advanced prosthetic and rehabilitation devices. Cipriani and colleagues created a shared control strategy for an EMG-controlled prosthetic hand, analyzing user interactions. They found that users operated the device well with hierarchical control and vibrotactile feedback but preferred less interactive strategies for effort-intensive tasks. This implies that while vibrotactile feedback boosts proprioception, its effectiveness varies by context and user preference [44]. Similarly, *D’Alonzo and Cipriani* [16] explored vibrotactile sensory substitution to induce the feeling of ownership of an alien hand in normally limbed subjects. The study found that synchronous, modality-conflicting visuo-tactile stimulation can induce self-attribution of a rubber hand. This suggests that sensory substitution may improve current haptic technologies in prosthetics. Advances have also addressed the sensory feedback gap in myoelectric prostheses. In a study by Raspopovic and colleagues (2019) [22], examined simultaneous tactile and proprioceptive feedback through intraneural stimulation, enabling transradial amputees to regain natural proprioceptive acuity and effectively discriminate object size and compliance. This study advances bidirectional bionic limbs, enhancing the sensory feedback and functionality of prosthetics [22]. D’Alonzo, Cipriani, and collaborators [17] Investigated if vibrotactile stimulation could induce the embodiment of a rubber hand in transradial amputees with phantom sensations. Results indicated that this method elicits a sense of body ownership, suggesting its potential integration into commercial prostheses to enhance user experience. Additionally, Cipriani with Valle and collaborators [21] compared intraneural stimulation strategies for delivering tactile information to amputees. Hybrid strategies with frequency and amplitude modulation offered the most natural tactile feedback, enhancing manual dexterity and reducing abnormal phantom limb perceptions. This highlights the need for proper sensory encoding strategies to improve prosthesis performance.

Finally, a study by Masiero, Cipriani, and their collaborators [23] addressed the challenge of evoking proprioceptive sensations with a pyrokinetic interface and implanted magnets. The research showcased a real-time system for high-precision tracking and vibrating multiple magnets, aiding advanced systems for studying human proprioception.

#### By country.

**Fig 2:**
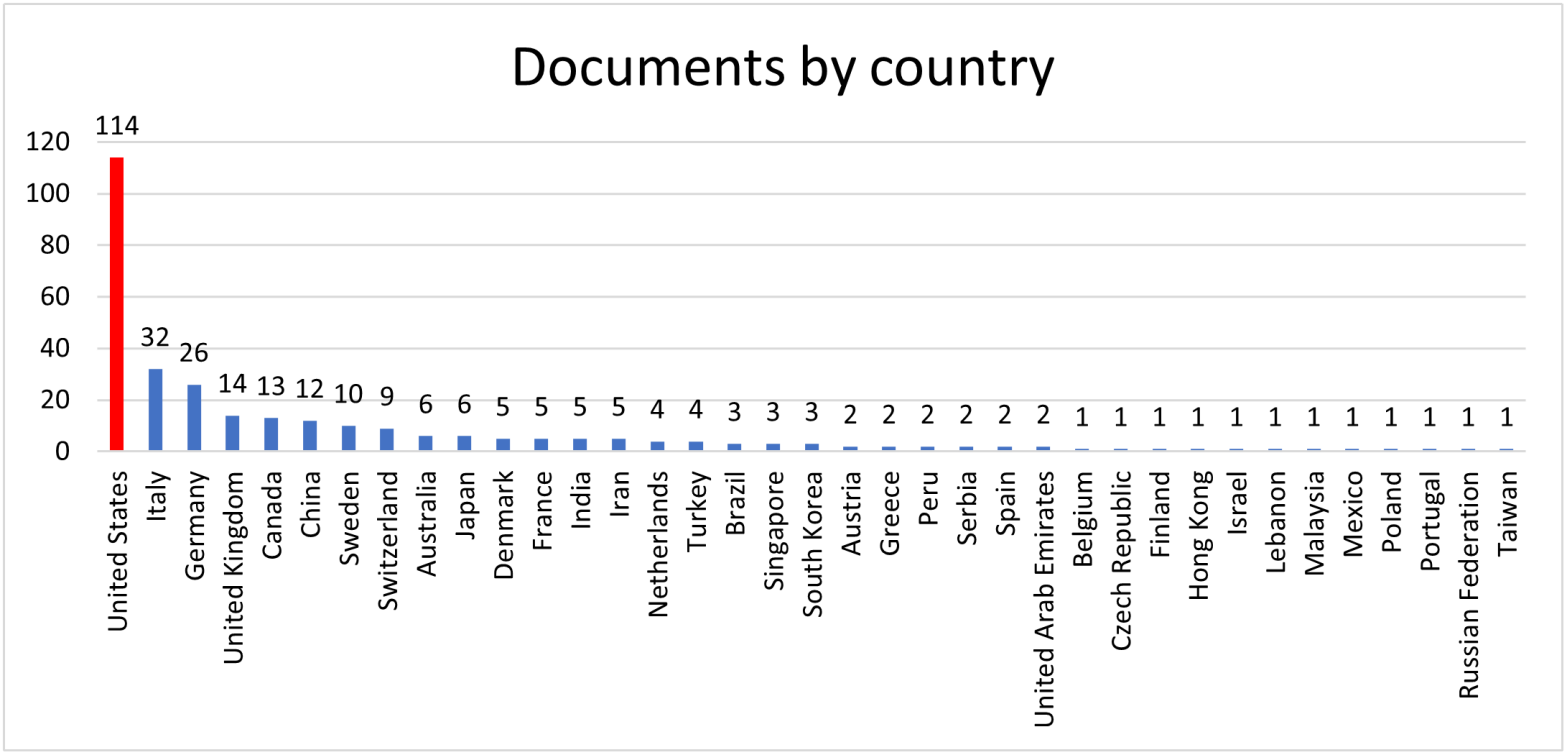
Chart by country and number of documents generated on the topic.

The allocation of documents across countries reveals the worldwide scope of research contributions regarding sensory feedback in prosthetics. The United States leads with 114 publications, followed by Italy (32), Germany (26), and the United Kingdom (UK) (14). Other contributors include Canada (13), China (12), and Sweden (10). Switzerland has nine, while Australia and Japan each contributed six. Denmark, France, India, and Iran add five publications, reflecting global diversity. The Netherlands and Türkiye each have four, with Brazil, Singapore, and South Korea contributing three each. Some countries have one publication. This participation highlights the collaborative and international nature of research in these fields. Several instances exist where a country’s single publication may result from a student collaborating during their time abroad. See Fig 2.

#### Evolution over the years.

Throughout the research, these articles examine proprioception and feedback related to prosthetics and neuroprosthetics. Several studies from 2005 to 2007 explored foundational elements of proprioception and its application in prosthetics. Wall III and Kentala in 2005, vibrotactile feedback was investigated for postural control in patients with deficits [54], while Farrell and colleagues [55] In 2005, the impact of static friction and backlash on powered prosthesis control was examined. Loeb and Tan [56] also in 2005 contributed to this foundational understanding by exploring biomimetic posture sensing for proprioception. Dhillon and colleagues (2005) presented a method for enhancing upper limb prosthetic functionality through direct neural control and sensory feedback. Using intrafascicular electrodes implanted in the peripheral nerves of amputees, the study demonstrated the feasibility of eliciting precise tactile and proprioceptive sensations while enabling motor control directly from neural signals [57]. Notably, Kuiken and colleagues (2007) introduced targeted reinnervation to redirect cutaneous sensation, enhancing sensory feedback in amputees [58].

Research from 2008 to 2011 highlights advancements in the technology and methodology for improving prosthetic control and feedback. Blank and colleagues [48] (2008) and Li and Kuiken [59] (2008) delved into the role of proprioceptive motion feedback in virtual hand prostheses and modeled prosthetic limb rotation control, respectively. In 2009, Wall III and colleagues [60] demonstrated that vibrotactile feedback on body tilt significantly enhances the Dynamic Gait Index in older adults, reducing fall risks and improving balance. Further developments include Horch and colleagues (2011) focusing on object discrimination using electrical stimulation of tactile and proprioceptive pathways [61].

From 2012 to 2015, the emphasis shifts towards integrating advanced neural interface systems and multi-electrode stimulation techniques. In 2012, Weber and colleagues [62] provided essential considerations for interfacing the somatosensory system to restore touch and proprioception. The authors recommend advancing the field by addressing knowledge gaps, improving technology, and exploring new somatosensory approaches and interfaces. Also, in 2012, Yang and colleagues [63] studied the LEAFS feedback system to enhance gait symmetry in transtibial amputees through auditory cues, showing promising rehabilitation results. In 2014, Mulvey and colleagues [64] studied transcutaneous electrical nerve stimulation (TENS) to enhance the embodiment of an artificial hand, indicating that somatosensory feedback may improve control in prosthetic users. Ramos-Murguialday and colleagues [65] explored brain–computer interface-based neuroprostheses with proprioceptive feedback. Concurrently, Dadarlat and colleagues [66] (2015) introduced a learning-based approach to artificial sensory feedback, optimizing sensory integration in neuroprosthetics.

From 2016 to 2021, significant progress has been made in practical applications and user experience with advanced prosthetics. For example, a study from Bensmaia [67] explores the neural coding principles underlying natural and bionic hands, focusing on replicating sensory inputs through artificial stimulation. While the primary emphasis is on tactile perception, the framework supports proprioception by integrating dynamic position sensing, which is essential for fine motor control.

In 2016, Plauche and colleagues [68] presented a haptic feedback system for above-knee prosthetic leg users, while in 2021, Wendelken and colleagues [15] demonstrated the restoration of motor control and sensation in amputees using Utah Slanted Electrode Arrays. Recent studies, such as those by Schiefer and colleagues [10] in 2018, who reported improvements in object identification tasks were reported through artificial tactile and proprioceptive feedback.

Marasco and colleagues (2018) explored the role of illusory movement perception in enhancing prosthetic motor control. Vibration reinnervated amputee muscles, inducing kinesthetic illusions of hand movement, allowing for more precise control of prosthetic devices with less visual feedback. This study showed that integrating movement perception into prosthetics improves functional outcomes and user experience, providing a new approach to sensory-motor deficits in neuroprosthetics [69].

And Patwardhan and colleagues (2019, 2020) [70,71] highlighted the importance of integrating proprioception into prosthetic control via *sonomyography*, suggesting that such feedback could improve the intuitiveness of prosthetic devices. Also in 2019, Battaglia and colleagues [72] explored using skin stretch haptic feedback to convey hand closure information in upper limb prostheses, reducing the need for visual attention and improving user control. Additionally, in 2019, Cuberovic and colleagues [13] emphasized the long-term home use of sensory-enabled prostheses, highlighting real-world applicability and user adaptation.

The most recent studies from 2021 to 2023 focus on refining sensory feedback and enhancing prosthetic control systems. Srinivasan and colleagues (2021) [73] explored agonist-antagonist myoneural interfaces for preserving joint function and perception in above-knee amputations.

Marasco and colleagues (2021) investigate integrating touch, kinesthesia, and motor control in bidirectional bionic prostheses. It shows that motor and sensory reinnervation enables users to achieve near-able-bodied function. Participants exhibited better sensory discrimination, enhanced visuomotor skills, and a stronger sense of prosthetic ownership. The study emphasizes sensory-motor fusion’s role in human-like prosthetics and provides a framework for assessing neuroprosthetic interventions [74].

In 2022, Magbagbeola and colleagues [75] investigated how vibration patterns can improve the perception of tactile information in prosthetic limbs, aiding in the long-term use of prosthetics and neuropathic pain management. Also, in 2022, Cha and colleagues [76] presented a closed-loop control system for robotic prosthetic hands, combining EMG-based intention recognition with proprioceptive feedback to enhance control.

Lima and Hammond [77] (2023) examined simultaneous rotary skin stretch and vibrotactile stimulation for haptically displayed proprioceptive feedback. In 2023, Bensmaia and colleagues [7] reviewed the restoration of sensory information via bionic hands, highlighting the integration of natural neural coding and artificial perception in contemporary prosthetic devices.

Cimolato and colleagues (2023) introduced ProprioStim, A biomimetic framework that restores proprioception in amputees using neurostimulation. This study combines neuromusculoskeletal models and electro-neural simulations to replicate essential afferent activity for joint position sensing. Validation with TENS showed significant improvements in proprioceptive accuracy compared to traditional methods. The authors highlight the system’s potential for real-time integration in neuroprostheses, enabling more natural and effective proprioceptive feedback in clinical applications [33].

Katic Secerovic and colleagues (2024) examined electrical stimulation’s effect on proprioceptive processing in spinal circuits. Using primate models, the authors found that simultaneous stimulation of cutaneous and proprioceptive afferents disrupts neural responses and alters proprioceptive integration. The study highlighted implications for neuroprosthetic design, advocating biomimetic strategies to preserve sensory-motor coherence and prevent harmful effects on perception and motor control [34].

### Our work in Mexico

In 2019, UNAM offered a bachelor’s in orthotics and prosthetics tailored to national needs. The university aims for research, design, and technological development in this transdisciplinary field. We have progressed with the AP proposal for prosthesis users, as detailed in our published work 2023 [78] Using a simple approach and an understandable methodology, the aim was and still is to integrate biomechatronic devices that imitate the sensory feedback lost due to amputation.

## Discussion

### Global research trends

The global research on AP in prosthetics is collaborative across various fields and regions. This trend aims to enhance the quality of life for those with limb amputations. Contributions are geographically diverse, primarily from the United States and Europe, with a growing presence of Asia representations.

### Geographical distribution of the contributions to research

The United States leads with 114 publications in prosthetic sensory feedback technologies, showcasing robust funding, and interdepartmental collaboration in its research institutions. Case Western Reserve University and Stanford University contribute significantly, reinforcing the U.S. position as an academic and clinical prosthetic technology leader.

Italy has 32 publications, indicating Europe’s strong representation in this field, mainly through major institutions like the BioRobotics Institute at *Scuola Superiore Sant’Anna*. Italy excels in robotics and advanced prosthetic control strategies, reflecting its focus on engineering and technological innovation. Other European countries, such as Germany, the UK, and Switzerland, contribute significantly, with institutions like ETH Zürich leading in prosthetic neuro-engineering solutions.

The most significant contributions come from Asia, particularly China, where research institutions engage in extensive international collaboration. Interest in neural interfaces and AI applications in prosthetics is growing, reflecting the globalization of technologies that foster inclusive research to address complex biomedical challenges.

### Institutional contributions and collaboration

Research distribution highlights the vital role of specialized institutions and universities in advancing AP. Sant’Anna Scuola Universitaria Superiore Pisa tops the list with 12 publications, followed by Northwestern University and MIT. These institutions foster innovation and unite biomedical engineering, neuroscience, and robotics experts.

Collaboration is high, with co-authored research reflecting cross-country partnerships. For instance, collaborations between European and U.S. universities significantly advance neural interfaces and sensory feedback mechanisms, addressing the complexities of creating intuitive, functional prosthetic devices.

### Restored proprioception and tactile feedback

Differentiating between restored proprioception and tactile feedback in prosthetic systems is crucial due to their distinct mechanisms. Proprioception restoration aims to replicate natural limb position, movement, and force sensing by stimulating muscle spindles and other proprioceptors. It provides precise feedback on joint angles and limb kinematics, which is critical for motor control without visual cues. Conversely, tactile feedback replicates sensations like pressure or vibration through skin stimulation. While proprioception aids subconscious limb integration for dynamic tasks, tactile feedback offers localized information on object interaction. Future research should combine these modalities in biomimetic frameworks, ensuring anatomically and contextually appropriate feedback to improve neuroprosthetic systems’ intuitiveness and effectiveness.

### Lower and upper limbs

The sensory feedback mechanisms in prostheses differ due to functional, sensory, and biomechanical needs. Upper limb prostheses utilize feedback systems for precise tasks like grasping, requiring detailed tactile and proprioceptive information on grip, finger position, and surface texture [18,69] . Electrotactile and vibrotactile feedback effectively convey this data. Conversely, lower limb prostheses focus on proprioceptive feedback for balance, gait coordination, and weight distribution, with feedback methods simulating joint angles, ground forces, and foot placement for stability [22,62]. Walking necessitates real-time feedback to adapt to terrain changes, emphasizing responsiveness in lower limb devices. While upper limb feedback aids fine motor skills, lower limbs prioritize gross motor coordination and stability. Future advancements should integrate task-specific sensory modalities for better activity transition and enhanced prosthetic user experience.

### Future directions in proprioceptive feedback and personalized prosthetics

We identified key areas needing research and technological development for personalized, efficient prosthetics. These insights stem from a thorough literature review and analyzed references:

*Advancing proprioceptive integration:* Despite progress in proprioceptive feedback systems (e.g., ProprioStim by Cimolato and colleagues (2023) [33]), current technologies struggle to provide high-resolution, real-time signals that are fully biomimetic. Research should refine neural interfaces, like intrafascicular electrodes [57], to ensure stable, long-term proprioceptive encoding with minimal interference from tactile feedback systems.

*User-specific adaptation needs:* A significant limitation noted in the literature is the inflexibility of prosthetic systems to meet individual users’ preferences. This involves adaptive algorithms that learn from user movements to adjust feedback parameters, as stated by Dadarlat and colleagues (2015) [66]. AI-based personalization systems are a vital research frontier.

*Improving multi-modal feedback integration:* Hybrid systems combining proprioceptive and tactile feedback [19,79] show promise but often lack anatomical congruency and integration. Research should focus on fusing tactile and proprioceptive cues to align sensory signals with natural motor and sensory pathways [4].

*Scalability and accessibility of advanced prosthetic systems:* The high cost and complexity of advanced proprioceptive systems (e.g., biomimetic frameworks by Bensmaia and colleagues (2023) [80]) restrict user accessibility. Future tech improvements should aim for scalable, cost-effective solutions that maintain functionality and sensory fidelity.

*Expanding functional testing beyond controlled environments:* Most studies by Marasco and colleagues (2018) [69] and Katic Secerovic and colleagues (2024) [34] validated proprioceptive feedback in laboratory conditions. However, gaps remain in assessing long-term usability, neural adaptation, and effectiveness in real-world scenarios. Therefore, functional testing should include diverse environments and daily tasks for practical applicability.

*Neural interface longevity and biocompatibility:* Page and colleagues (2021) [8] highlight that long-term neural interface use faces challenges like electrode degradation, immune response, and signal drift. Research on biocompatible materials, like soft neural interfaces, may improve prosthetic durability and safety.

*Improving lower limb proprioception:* Lower-limb prosthetics need more focus on proprioceptive cues for balance and gait, unlike the upper limbs. Innovations like phase-based sensory systems [68] should be further explored to fill these gaps.

## Conclusion

The review evaluates AP innovations crucial for restoring amputees’ sensory feedback. It highlights technological advances seeking to replicate natural proprioceptive systems, focusing on the involved neural mechanisms and biomechatronics. Following amputation, the loss of proprioception disrupts the connection between the prosthetic limb and the user’s movement perception, causing challenges in motor control and task execution.

Global efforts in AP development for prosthetics have resulted in innovations like neural interfaces, advanced controls, and AI, enhancing functionality and user experience. These advancements aim to restore amputees’ quality of life by embedding proprioceptive feedback in designs. Neural interfaces connect with the nervous system to improve the natural control of prosthetic limbs.

For lower-limb prosthetics, proprioceptive feedback is crucial for balance and gait, with technologies like vibrotactile and electrotactile feedback improving mobility and stability. Despite advancements, ongoing research and technological refinement, are necessary to meet the increasing demand for more personalized and efficient prosthetic solutions, as described in the section “Future directions in proprioceptive feedback and personalized prosthetics.”

Artificial restoration of proprioception is essential for prosthetic embodiment, enhancing both functionality and acceptance. Proprioceptive feedback fosters a sense of ownership and agency, reducing reliance on visual cues and improving motor control. By seamlessly integrating the prosthetic into the body schema, users experience greater confidence, better performance, and improved quality of life.

## Supporting information

S1 Data Mendeley database. (RIS)
